# 

**Published:** 1886-03

**Authors:** 


### CONTENTS.

ORIGINAL ARTICLES—Alcoholism—Treatment of Orchitis—Puerperal Eclampsia—Enlarged Spleen Treated by Ergot Hypodermatically .................. 393–407

CULLINGS FROM CONTEMPORARIES—Novel Method of Entering Bladder—Inoculation Gone Mad .......... 408–409

CORRESPONDENCE—Our New York Letter—Von Brun's Bandage—A Rare Case.......................... 410–413

ABSTRACTS FROM FOREIGN EXCHANGES................ 413

SOCIETY NOTES—American Medical Association - Eighteenth Annual Convention T. S. M. A.—Biography of Contemporary Physicians of Texas Association of American Medical Editors—The Physicians' Mutual Benefit Association of Texas—Prize Essays Texas State Medical Association—Circular—Travis County Medical Association - Delegates ......... 414–420

EDITORIAL—The Tug of War—How Does Journalism Aid the Development of the Medical Profession ; How has it Benefitted the Profession of Texas ?—"Ye Festive Bolter" ......................... 421–427

EDITORIAL NOTES - Homœopathic Grammar—The Nameless Knight—Texas Carries off the Honors—The Association Captured—Who?—The (Self) Elect, etc—Married .............................. 429–434

BIBLIOGRAPHY.................................. 434–438

ADVERTISERS' NOTICES.......................... 438–439

